# Supplementary figures and images for: Regional variations in primary percutaneous coronary intervention for acute myocardial infarction patients: A trajectory analysis using the national claims database in Japan
Source: PLoS One. 2024 Oct 22;19(10):e0312248. doi: 10.1371/journal.pone.0312248 (PMC11495596; doi:10.1371/journal.pone.0312248)

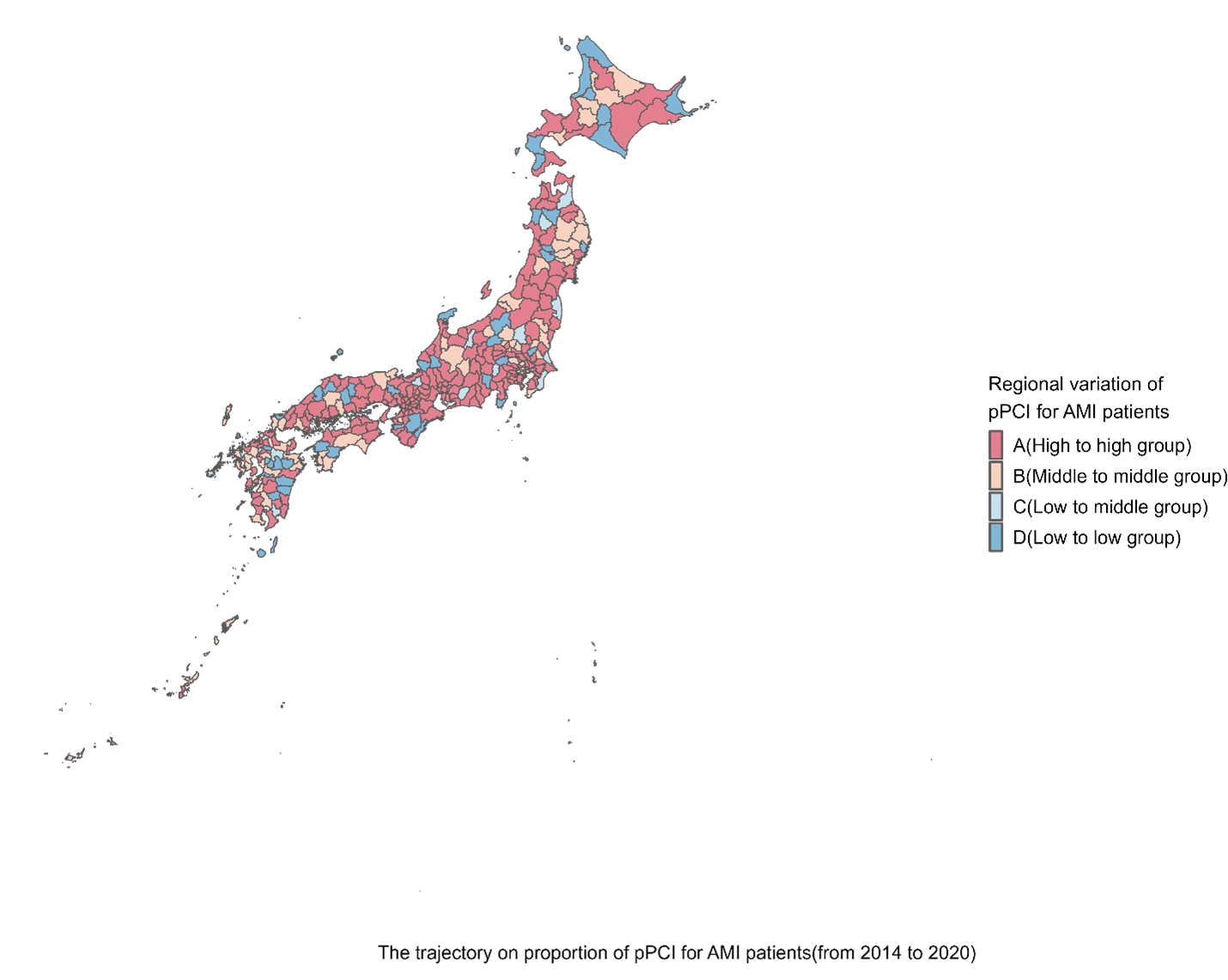

Supplement: S1 Fig — Group A (high to high) indicated that the proportion of pPCI for AMI patients was high from 2014 to 2020, group B (middle to middle) indicated that the proportion of pPCI for AMI patients was moderate from 2014 to 2020, group C (low to middle) indicated that the proportion of pPCI for AMI patients had increased from 2014 to 2020, and group D (low to low) indicated that the proportion of pPCI for AMI patients was low from 2014 to 2020. (TIF) [file pone.0312248.s001.tif]

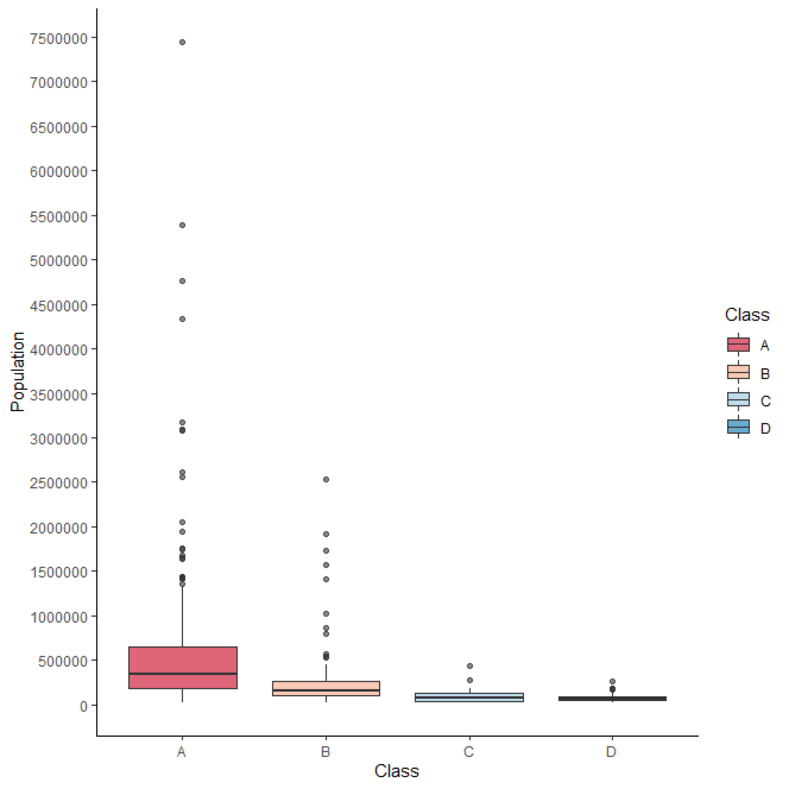

Supplement: S2 Fig — (TIF) [file pone.0312248.s002.tif]

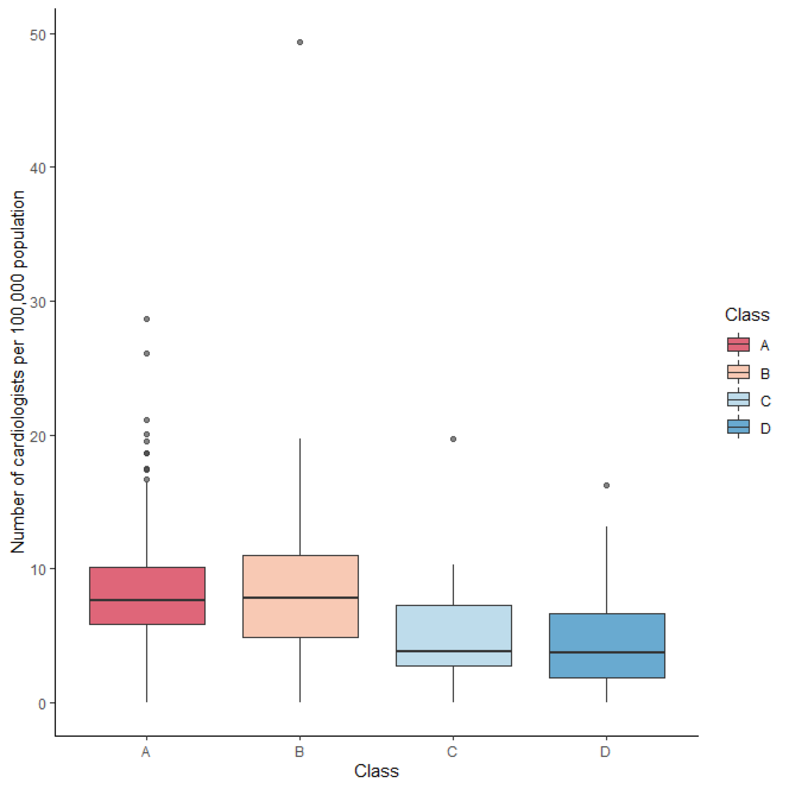

Supplement: S3 Fig — (TIF) [file pone.0312248.s003.tif]

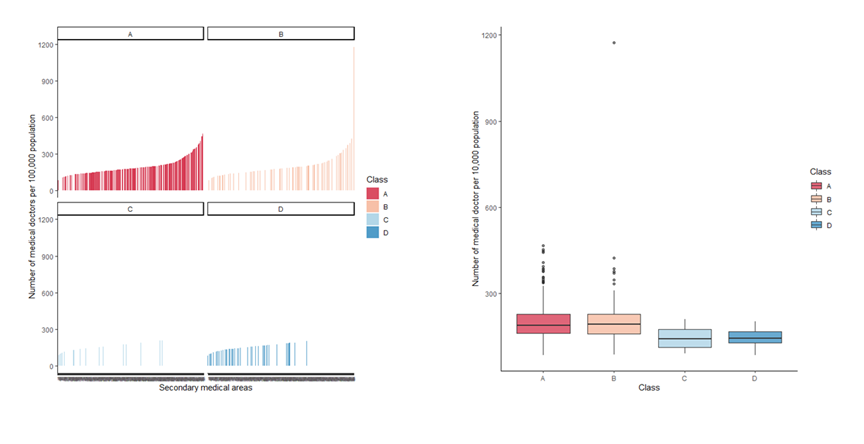

Supplement: S4 Fig — (TIF) [file pone.0312248.s004.tif]

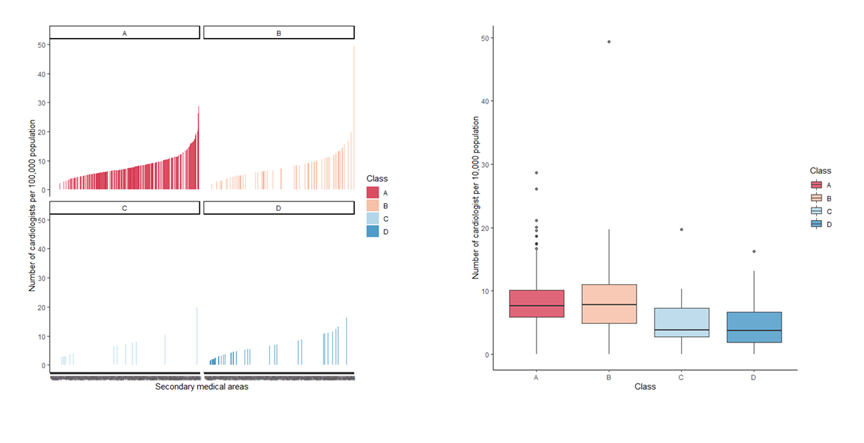

Supplement: S5 Fig — (TIF) [file pone.0312248.s005.tif]

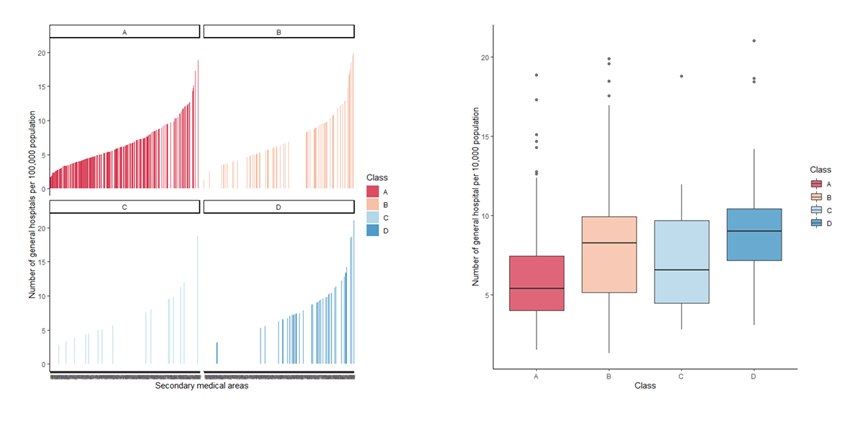

Supplement: S6 Fig — (TIF) [file pone.0312248.s006.tif]

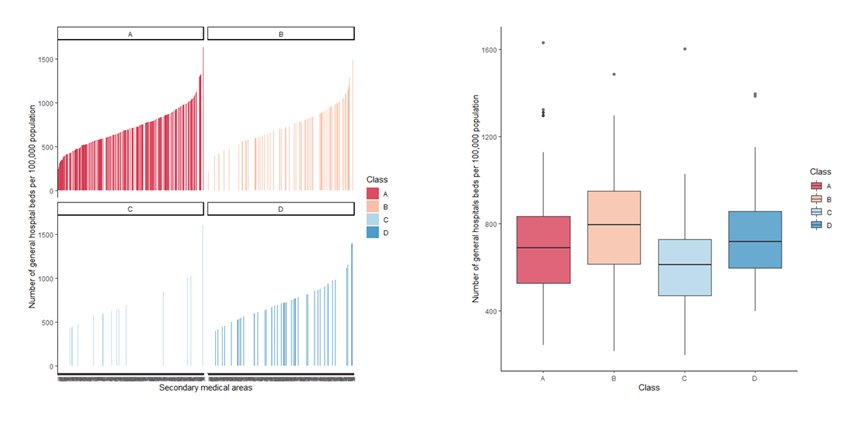

Supplement: S7 Fig — (TIF) [file pone.0312248.s007.tif]
